# A dynamic recovery failure index after acute type A aortic dissection surgery

**DOI:** 10.1080/07853890.2026.2724602

**Published:** 2026-09-04

**Authors:** Shuaishuai Yuan, Lingling Li, Minxin Wei, Zanxin Wang, Rui Zhang, Yue Ma, Zijian Wang, Chao Tao, Zenan Zhou, Xiantao Liu, Yi Lu, Qi An, Fengqing Liu, Guiqiong Fu, Rongfeng Yang, Peijun Li

**Affiliations:** ^a^ Division of Cardiovascular Intensive Care (C-ICU), Cardiac and Vascular Center, The University of Hong Kong-Shenzhen Hospital, Guangdong Province, China; ^b^ Shenzhen Maternity and Child Healthcare Hospital, Southern Medical University, Shenzhen, Guangdong Province, China; ^c^ Division of Cardiovascular and Vascular Surgery, Cardiac and Vascular Center, The University of Hong Kong-Shenzhen Hospital, Guangdong Province, China

**Keywords:** Acute type A aortic dissection, severe organ dysfunction, inflammation, perfusion, coagulation, lactate clearance, platelet decline, risk restratification, temporal validation

## Abstract

**Background:**

Acute type A aortic dissection (ATAAD) is associated with a high risk of postoperative severe organ dysfunction. Preoperative risk scores alone may not capture early postoperative pathophysiological recovery. This study aimed to develop and temporally validate a dynamic inflammation–perfusion–coagulation recovery failure index (dIPC-RFI), based on routinely available preoperative, immediate postoperative, and 24-hour postoperative laboratory and blood gas variables, for risk restratification of severe organ dysfunction beyond 24 h after ATAAD surgery.

**Methods:**

Patients with ATAAD who underwent emergency surgical repair at The University of Hong Kong-Shenzhen Hospital between August 2021 and December 2025 were retrospectively included. Postoperative 24 h was used as the fixed risk-assessment starting point; only patients who were alive, free from the primary outcome, and had complete variables required for dIPC-RFI assessment at 24 h were included. Patients were split chronologically into a derivation cohort (first 70%) and a temporal validation cohort (last 30%). The primary outcome was severe organ dysfunction occurring after 24 h until discharge or within 30 days, defined as any of the following: continuous renal replacement therapy, mechanical ventilation for ≥72 h, neurological complications, multiple organ dysfunction syndrome, in-hospital death after 24 h or 30-day death, intra-aortic balloon pump support, extracorporeal membrane oxygenation, or tracheostomy. dIPC-RFI consisted of three domains—inflammation, perfusion, and coagulation—each including one static 24-hour variable and one dynamic change variable. Cutoffs were derived using receiver operating characteristic curves and the Youden index in the derivation cohort and then fixed for the validation cohort. Model performance was assessed using the area under the receiver operating characteristic curve (AUC), Brier score, calibration curve, calibration intercept and slope, and decision curve analysis.

**Results:**

A total of 330 patients were included, of whom 134 (40.6%) developed severe organ dysfunction after 24 h. Event rates were 39.0% in the derivation cohort and 44.4% in the temporal validation cohort (*p* = 0.392). In the validation cohort, dIPC-RFI achieved an AUC of 0.873 (95% CI, 0.794–0.935) and a Brier score of 0.1387, outperforming the simplified clinical model (AUC = 0.762) and the extended clinical model (AUC = 0.735). The combined model integrating the simplified clinical model and dIPC-RFI further improved discrimination (AUC = 0.908) and reduced the Brier score to 0.1243. Component-specific analyses showed acceptable discrimination for major components with sufficient validation events, including CRRT, prolonged mechanical ventilation, neurological complications, and MODS component. The no-CRRT sensitivity analysis and the death-or-MODS sensitivity analysis supported the robustness of the main findings.

**Conclusions:**

dIPC-RFI provides a simple 24-hour postoperative framework for identifying incomplete recovery across inflammation, perfusion, and coagulation after ATAAD surgery. It may help ICU teams identify patients who require intensified monitoring, repeated lactate and coagulation assessment, early multidisciplinary review, and preparation for organ support. dIPC-RFI should be used as a risk-restratification aid rather than a stand-alone treatment trigger, and prospective multicenter validation is required before routine implementation.

## Key messages

dIPC-RFI integrates routinely available inflammatory, perfusion, and coagulation variables measured before surgery, immediately after surgery, and 24 hours after surgery.

In a temporal validation cohort, dIPC-RFI showed good discrimination for severe organ dysfunction occurring beyond 24 hours after ATAAD surgery and provided clear low-, intermediate-, and high-risk strata.

The score is intended for postoperative 24-hour bedside risk restratification, not for preoperative decision-making, and requires external validation before routine clinical implementation.

## Graphical abstract

**Figure:**
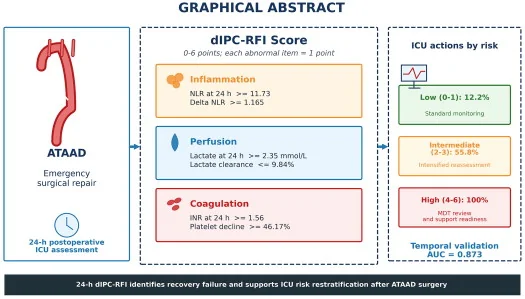

## Introduction

Acute type A aortic dissection is a rapidly progressive and life-threatening cardiovascular emergency. Emergency surgical repair remains the mainstay of treatment; however, even after successful aortic repair, patients remain vulnerable to postoperative severe organ dysfunction because of preoperative malperfusion, cardiopulmonary bypass injury, deep hypothermic circulatory arrest, ischemia-reperfusion injury, amplified systemic inflammation, and coagulation disturbances. Previous studies have shown that postoperative organ dysfunction, acute kidney injury, respiratory failure, circulatory failure, and neurological complications are closely associated with in-hospital mortality, prolonged intensive care unit stay, and poor long-term outcomes [1–3]. Early identification of high-risk patients after surgery is therefore important for intensified monitoring and preparation for organ support.

Currently used tools, such as EuroSCORE II and the German Registry for Acute Type A Aortic Dissection (GERAADA) score, mainly estimate operative mortality risk [4,5]. The Sequential Organ Failure Assessment (SOFA) score can describe organ dysfunction but requires multiple clinical variables and is more often used to assess the severity of dysfunction after it has occurred [6]. In recent years, the neutrophil-to-lymphocyte ratio (NLR), lactate and lactate clearance, coagulation abnormalities, and thrombocytopenia have been associated with adverse outcomes after aortic dissection or major cardiovascular surgery [7–14]. Nevertheless, most previous studies focused on single biomarkers or single-organ outcomes, and comprehensive assessment of dynamic recovery failure across inflammation, perfusion, and coagulation remains insufficient.

Against this background, we positioned the present study as the development and temporal validation of a postoperative risk restratification tool for severe organ dysfunction beyond 24 h after surgery, rather than as a purely preoperative prediction model or an interventional study. We used postoperative 24 h as a fixed risk-assessment starting point and used only information available at or before that time to stratify the risk of severe organ dysfunction occurring after 24 h. We proposed dIPC-RFI to integrate serial changes from the preoperative period to immediate postoperative assessment and postoperative 24 h, aiming to quantify early recovery failure across inflammation, perfusion, and coagulation systems. We then evaluated its value for risk stratification after ATAAD surgery using temporal split validation.

## Methods

### Study population

We retrospectively included patients with acute type A aortic dissection who underwent emergency surgical repair at The University of Hong Kong-Shenzhen Hospital between August 2021 and December 2025. Patients were ordered by the date of surgery and split chronologically, with the first 70% assigned to the derivation cohort and the last 30% assigned to the temporal validation cohort.

Inclusion criteria were: age ≥18 years; diagnosis of ATAAD confirmed by computed tomography angiography, echocardiography, or intraoperative findings; receipt of emergency surgical treatment; availability of key preoperative, immediate postoperative, and postoperative 24-hour laboratory indicators; and clearly documented postoperative outcomes. Exclusion criteria were: preoperative long-term dialysis; end-stage malignant disease, severe chronic liver failure, or chronic respiratory failure before surgery; intraoperative death; death within 24 h after surgery; occurrence of the primary outcome before postoperative 24 h; severely missing perioperative data; repeated admission; or repeated surgery.

To avoid temporal ambiguity between postoperative 24-hour variables and outcomes that had already occurred, postoperative 24 h was used as the fixed risk-assessment starting point. Only clinical and laboratory information available at or before postoperative 24 h was used to evaluate the risk of severe organ dysfunction after 24 h. All primary outcome events were confirmed to occur after completion of dIPC-RFI assessment.

This retrospective study was approved by the Ethics Committee of The University of Hong Kong-Shenzhen Hospital (approval No. [2026]162). The requirement for informed consent was waived by the ethics committee because of the retrospective nature of the study and the use of de-identified clinical data. The study was conducted in accordance with the Declaration of Helsinki. The authors declare no competing interests.

### Data collection

We collected demographic characteristics, preoperative clinical status, operative variables, laboratory variables from the preoperative period to immediate postoperative assessment and postoperative 24 h, and postoperative outcomes. Demographic variables included age, sex, body mass index (BMI), hypertension, diabetes, smoking history, and alcohol use. Preoperative clinical status included cerebral malperfusion and pericardial effusion. Operative variables included surgical procedure, cardiopulmonary bypass time, aortic cross-clamp time, operative duration, red blood cell transfusion, plasma transfusion, platelet transfusion, and bleeding volume. Laboratory variables were organized at three time points: T0, preoperative; T1, immediate postoperative or immediately after admission to the cardiac intensive care unit; and T2, postoperative 24 h. Laboratory indicators were grouped into inflammatory, perfusion, and coagulation domains. Inflammatory variables included white blood cell count, neutrophil count, lymphocyte count, and NLR. Perfusion variables included lactate, lactate clearance, pH, and base excess. Coagulation variables included platelet count, international normalized ratio (INR), activated partial thromboplastin time (APTT), fibrinogen, and D-dimer.

### Primary outcome

The primary outcome was severe organ dysfunction occurring after postoperative 24 h. It was defined as the occurrence of any of the following events after postoperative 24 h and before discharge or within 30 days after surgery: continuous renal replacement therapy (CRRT), mechanical ventilation for ≥72 h, neurological complications, multiple organ dysfunction syndrome (MODS), in-hospital death after postoperative 24 h or death within 30 days, intra-aortic balloon pump (IABP) support, extracorporeal membrane oxygenation (ECMO), or tracheostomy. All primary outcome events were confirmed to occur after completion of dIPC-RFI scoring.

### Dynamic variables in the dIPC-RFI model

In the derivation cohort, we compared the AUCs of all available preoperative-to-24-hour inflammatory, perfusion, and coagulation variables, including absolute values, differences, and ratios. We did not force all dynamic variables to use the same mathematical form. Instead, we selected clinically meaningful dynamic indicators according to the pathophysiology of inflammation, perfusion, and coagulation systems. After comparison of candidate indicators, one static variable and one dynamic variable were selected from each domain: NLR at postoperative 24 h and delta NLR for inflammation, lactate at postoperative 24 h and lactate clearance for perfusion, and INR at postoperative 24 h and platelet decline rate for coagulation. Delta NLR was defined as NLR at postoperative 24 h minus preoperative NLR. Lactate clearance was defined as (immediate postoperative lactate–postoperative 24-hour lactate)/immediate postoperative lactate x 100%. Platelet decline rate was defined as (preoperative platelet count - postoperative 24-hour platelet count)/preoperative platelet count x 100%.

### Construction of dIPC-RFI and risk strata

dIPC-RFI consisted of three domains—inflammation, perfusion, and coagulation—each including one static postoperative 24-hour variable and one dynamic change variable, for a total of six items. In the derivation cohort, all cutoffs were determined using ROC curves and the Youden index with severe organ dysfunction beyond 24 h after surgery as the outcome. Each abnormal item was assigned 1 point, yielding a total score ranging from 0 to 6. Patients were classified into low recovery failure (0–1 points), intermediate recovery failure (2–3 points), and high recovery failure (4–6 points) groups.

To assess whether dichotomizing variables into a point score caused substantial information loss, we constructed a continuous-variable model as a sensitivity analysis. In this model, NLR at postoperative 24 h, delta NLR, lactate at postoperative 24 h, lactate clearance, INR at postoperative 24 h, and platelet decline rate were entered directly as continuous variables into a logistic regression model, and performance was evaluated in the temporal validation cohort.

### Unified rate-of-change version: dIPC-RFI-rate

To evaluate the influence of dynamic-variable definition on model performance, we further constructed a unified rate-of-change version, dIPC-RFI-rate, as a sensitivity analysis. The unified change rate was defined as the percentage change from preoperative assessment to postoperative 24 h: (T2 - T0)/T0 x 100%. Because greater platelet decline indicates higher risk, platelet decline was still defined as (preoperative platelet count - postoperative 24-hour platelet count)/preoperative platelet count x 100%. dIPC-RFI-rate included NLR at postoperative 24 h, NLR change rate, lactate at postoperative 24 h, lactate change rate, INR at postoperative 24 h, and platelet decline rate.

### Temporal validation and model development

The database was ordered chronologically by surgical date. Temporal split validation was performed by assigning the first 70% of patients to the derivation cohort and the last 30% to the temporal validation cohort. The derivation cohort was used to determine cutoffs and develop models, whereas the validation cohort was used to assess model performance. All model variables were restricted to information available at or before postoperative 24 h, and the target outcome was severe organ dysfunction occurring after 24 h.

Based on clinical interpretability, routine availability, and model parsimony, age, cardiopulmonary bypass time, and red blood cell transfusion were selected to construct a simplified clinical model, representing baseline patient risk, operative/cardiopulmonary bypass burden, and perioperative transfusion burden, respectively. The purpose of this model was not to build the most complex clinical prediction model, but to provide a simple, stable, and clinically interpretable comparator to evaluate whether dIPC-RFI provided incremental risk stratification beyond routine clinical information. To avoid model instability and overfitting due to excessive variables, the simplified clinical model did not include a large number of preoperative and intraoperative variables.

Four primary models were constructed: a simplified clinical model (age, cardiopulmonary bypass time, and red blood cell transfusion), the dIPC-RFI model, the continuous-variable model, and the combined model (simplified clinical model plus dIPC-RFI).

To address the possibility of under-adjustment in the simplified clinical model and because perioperative bleeding and transfusion could influence postoperative coagulation indicators and organ dysfunction, we constructed an extended clinical model as a supplementary analysis. The extended clinical model specifically included plasma transfusion, platelet transfusion, and bleeding volume to evaluate the incremental risk stratification value of dIPC-RFI after adjustment for bleeding/transfusion-related factors. The extended clinical model included age, sex, body mass index, hypertension, diabetes, cerebral malperfusion, pericardial effusion, cardiopulmonary bypass time, aortic cross-clamp time, operative duration, red blood cell transfusion, plasma transfusion, platelet transfusion, and bleeding volume. Model performance was compared before and after addition of dIPC-RFI.

### Statistical analysis

Continuous variables were summarized as mean ± standard deviation or median (interquartile range), depending on distribution. Categorical variables were summarized as number and percentage. Between-group comparisons were performed using the t test, Mann–Whitney U test, chi-square test, or Fisher exact test, as appropriate. Postoperative 24 h was used as the fixed risk-assessment starting point; dIPC-RFI and combined models were built using variables available at or before postoperative 24 h, and the target outcome was severe organ dysfunction occurring after postoperative 24 h. Model discrimination was assessed using the AUC. Overall prediction error was assessed using the Brier score. Calibration was evaluated using calibration curves, calibration intercepts, and calibration slopes. A two-sided P value <0.05 was considered statistically significant. Statistical analyses were primarily performed using R software. Bootstrap resampling with 2000 repetitions was used to calculate 95% confidence intervals. DeLong tests were used to compare AUCs between models [15]. Logistic recalibration was used to evaluate improvement in probability calibration in the validation cohort [16]. Decision curve analysis was performed using the dcurves package to assess clinical net benefit across risk thresholds [17]. Reporting of this study was guided by the TRIPOD statement for clinical prediction models [18].

To address the potential optimism introduced by data-driven cutoff selection, all dIPC-RFI cutoffs were derived only in the derivation cohort using the Youden index and were then fixed before temporal validation. Component-specific AUC analyses were performed in the temporal validation cohort to assess the heterogeneous components of the composite endpoint. Variance inflation factors were calculated for the continuous-variable model and the extended clinical models to assess multicollinearity.

Missing data were handled using a complete-case approach for variables required for dIPC-RFI assessment and model evaluation. Because dIPC-RFI requires serial preoperative, immediate postoperative, and postoperative 24-hour variables, patients without the required variables or without clearly documented outcomes were not included in the primary analysis. Missingness of key variables was summarized in the Supplementary Appendix.

## Results

### Baseline and cohort characteristics

A total of 330 patients with ATAAD who were alive and assessable at the fixed postoperative 24-hour time point were included. According to chronological surgical date, 231 patients were assigned to the derivation cohort and 99 patients to the temporal validation cohort. Severe organ dysfunction beyond 24 h occurred in 134 patients (40.6%) overall, including 90 patients (39.0%) in the derivation cohort and 44 patients (44.4%) in the temporal validation cohort (*p* = 0.392). The two cohorts were generally comparable in age, sex, body mass index, cardiopulmonary bypass time, aortic cross-clamp time, operative duration, and primary outcome incidence. All variables used for dIPC-RFI scoring were measured at or before the postoperative 24-hour assessment point, and all components of the primary outcome occurred after completion of dIPC-RFI assessment. Baseline and cohort characteristics are summarized in Table 1.

**Table 1.** Baseline and cohort characteristics.

Variable	Overall (*N* = 330)	Derivation cohort (*N* = 231)	Validation cohort (*N* = 99)	*P* value
Male sex	281 (85.2%)	196 (84.8%)	85 (85.9%)	0.867
Age, years	47.5 [38.2–57]	47 [38–56]	48 [40.5–60]	0.067
BMI	25.1 [22.8–27.8]	24.8 [22.6–27.7]	26.1 [23.3–28.5]	0.063
Smoking	105 (31.8%)	73 (31.6%)	32 (32.3%)	0.898
Alcohol use	56 (17%)	39 (16.9%)	17 (17.2%)	1.00
Coronary artery disease	15 (4.5%)	9 (3.9%)	6 (6.1%)	0.396
Hypertension	232 (70.3%)	158 (68.4%)	74 (74.7%)	0.293
Diabetes mellitus	14 (4.2%)	11 (4.8%)	3 (3%)	0.566
Hyperlipidemia	13 (3.9%)	12 (5.2%)	1 (1%)	0.119
Preoperative cerebral malperfusion	99 (30%)	67 (29%)	32 (32.3%)	0.600
Preoperative pericardial effusion	124 (37.6%)	86 (37.2%)	38 (38.4%)	0.586
Postoperative paraplegia	49 (14.8%)	37 (16%)	12 (12.1%)	0.403
Postoperative CRRT	46 (13.9%)	36 (15.6%)	10 (10.1%)	0.226
Cardiopulmonary bypass time, min	170.5 [143.2–202]	173 [146–203.5]	165 [142–202]	0.235
Aortic cross-clamp time, min	110 [91–134]	112 [93–132]	106 [84–137]	0.265
Operative duration, h	6.3 [5.4–7.4]	6.3 [5.4–7.3]	6.4 [5.3–7.4]	0.488
Red blood cell transfusion, U	0 [0–2]	0 [0–2]	0 [0–4]	0.158
Plasma transfusion, mL	0 [0–0]	0 [0–0]	0 [0–0]	0.028*
Platelet transfusion, therapeutic doses	2 [2–2]	2 [2–2]	2 [2–2]	0.621
Bleeding volume, mL	400 [300–500]	400 [300–500]	400 [300–600]	0.011*
CICU stay, days	7 [5–10]	6 [5–10]	7 [5–11]	0.055
Total hospital stay, days	21 [16–28]	21 [16–29]	20 [16–26]	0.353
Severe organ dysfunction beyond 24 h	134 (40.6%)	90 (39%)	44 (44.4%)	0.392

*Note:* Continuous variables are presented as median [IQR]. *P* values were derived from Wilcoxon rank-sum tests or Fisher exact tests as appropriate.

### dIPC-RFI components and cutoffs

The final dIPC-RFI consisted of six routinely available variables representing three recovery-failure domains: inflammation, perfusion, and coagulation. The inflammatory domain included NLR at postoperative 24 h and the change in NLR from the preoperative value to postoperative 24 h. The perfusion domain included lactate at postoperative 24 h and lactate clearance from immediate postoperative assessment to postoperative 24 h. The coagulation domain included INR at postoperative 24 h and platelet decline from the preoperative value to postoperative 24 h. In the derivation cohort, the selected cutoffs were NLR at postoperative 24 h > =11.73, delta NLR > =1.165, lactate at postoperative 24 h > =2.35 mmol/L, lactate clearance < =9.84%, INR at postoperative 24 h > =1.56, and platelet decline > =46.17%. Each abnormal item was assigned 1 point, yielding a total score from 0 to 6. The dIPC-RFI components and derivation-cohort cutoffs are presented in Table 2.

**Table 2.** Components of the dIPC-RFI model and derivation-cohort cutoffs.

Variable	Derivation AUC	Cutoff	Direction	Sensitivity	Specificity	Score
NLR at postoperative 24 h	0.802	11.731	> =	0.756	0.794	1
Delta NLR from preoperative to 24 h	0.801	1.165	> =	0.822	0.652	1
Lactate at postoperative 24 h, mmol/L	0.808	2.350	> =	0.589	0.915	1
Lactate clearance, %	0.699	9.839	< =	0.633	0.794	1
INR at postoperative 24 h	0.638	1.560	> =	0.367	0.950	1
Platelet decline rate, %	0.680	46.174	> =	0.511	0.816	1

*Note:* Each abnormal item was assigned 1 point. Lactate clearance was scored as abnormal when < = cutoff; other items were scored as abnormal when > = cutoff.

### Risk strata and recovery-failure phenotypes

Patients were classified into low-score (0–1), intermediate-score (2–3), and high-score (4–6) groups. In the temporal validation cohort, the event rates were 12.2%, 55.8%, and 100.0%, respectively, showing a clear dose-response relationship. Three-system recovery-failure phenotypes also showed an increasing risk gradient, supporting the clinical interpretability of the inflammation-perfusion-coagulation framework. Detailed strata and phenotype tables are provided in the Supplementary Appendix (Tables S2–S3).

**Table 3.** Model performance in the temporal validation cohort.

Model	Validation N	Events	AUC (95% CI)	Brier score (95% CI)	Calibration intercept (95% CI)	Calibration slope (95% CI)
Simplified clinical	99	44	0.762 (0.663–0.859)	0.2029 (0.1744–0.2328)	0.749 (0.157–1.566)	1.985 (1.180–3.441)
dIPC-RFI	99	44	0.873 (0.794–0.935)	0.1387 (0.0973–0.1834)	0.810 (0.279–1.682)	1.228 (0.809–2.209)
Combined	99	44	0.908 (0.843–0.962)	0.1243 (0.0866–0.1685)	0.938 (0.356–1.866)	1.308 (0.915–2.191)
Continuous-variable	99	44	0.878 (0.810–0.933)	0.1519 (0.1074–0.2026)	0.097 (−0.464-0.825)	0.801 (0.558–1.250)
Extended clinical	99	44	0.735 (0.628–0.827)	0.2048 (0.1763–0.2357)	0.555 (0.074–1.125)	1.667 (1.011–2.676)
Extended clinical + dIPC-RFI	99	44	0.908 (0.842–0.962)	0.1254 (0.0843–0.1676)	0.973 (0.380–1.905)	1.336 (0.904–2.353)
dIPC-RFI-rate	99	44	0.885 (0.815–0.946)	0.1395 (0.0952–0.1865)	1.093 (0.494–2.086)	1.224 (0.844–2.136)

*Note:* Confidence intervals were calculated using 2000 bootstrap resamples. The extended clinical model was interpreted as a supplementary adjustment model.

### Model performance and multicollinearity assessment

In the temporal validation cohort, dIPC-RFI showed good discrimination for severe organ dysfunction beyond postoperative 24 h, with an AUC of 0.873 (95% CI, 0.794–0.935) and a Brier score of 0.1387 (95% CI, 0.0973–0.1834). The simplified clinical model had an AUC of 0.762 (95% CI, 0.663–0.859) and a Brier score of 0.2029 (95% CI, 0.1744–0.2328). Combining dIPC-RFI with the simplified clinical model further improved performance, with an AUC of 0.908 (95% CI, 0.843–0.962) and a Brier score of 0.1243 (95% CI, 0.0866–0.1685). The continuous-variable model showed a similar AUC of 0.878 (95% CI, 0.810–0.933), suggesting that conversion of the six variables into a simple bedside point score did not cause substantial loss of discriminatory information.

**Figure 1.**
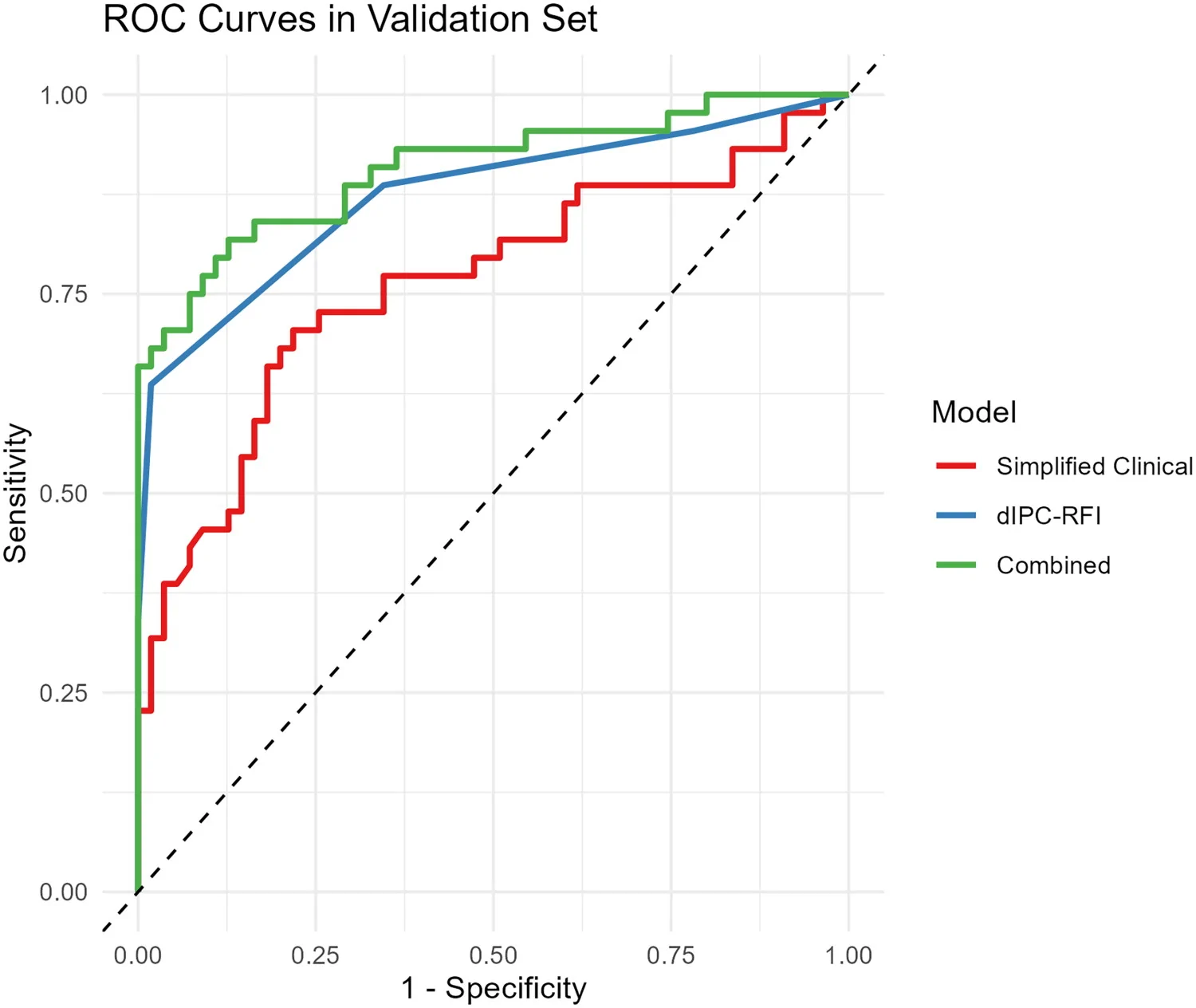
Receiver operating characteristic curves of different models for predicting severe organ dysfunction beyond 24 h after surgery in the temporal validation cohort. The red line indicates the simplified clinical model, the blue line indicates the dIPC-RFI model, and the green line indicates the combined model. The dashed line indicates the reference line of no predictive value. The combined model had the highest AUC, followed by dIPC-RFI and the simplified clinical model, suggesting that addition of dIPC-RFI improved model discrimination.

To formally evaluate multicollinearity, variance inflation factors were calculated for the continuous-variable model and the extended clinical models. The maximum VIF in the continuous-variable model was 1.574, indicating no substantial multicollinearity among the six continuous dIPC-RFI components. In the extended clinical model, the maximum VIF was 8.173, and after adding dIPC-RFI it was 8.501. The variables with VIF values greater than 5 were mainly cardiopulmonary bypass time and aortic cross-clamp time, reflecting expected clinical correlation among operative-time variables. Therefore, the extended clinical model was interpreted as a supplementary adjustment model rather than the primary comparator.Model performance metrics in the temporal validation cohort are shown in Table 3. Figure 1 presents the receiver operating characteristic curves for the simplified clinical model, dIPC-RFI, and the combined model in the temporal validation cohort.

To formally evaluate multicollinearity, variance inflation factors were calculated for the continuous-variable model and the extended clinical models. The maximum VIF in the continuous-variable model was 1.574, indicating no substantial multicollinearity among the six continuous dIPC-RFI components. In the extended clinical model, the maximum VIF was 8.173, and after adding dIPC-RFI it was 8.501. The variables with VIF values greater than 5 were mainly cardiopulmonary bypass time and aortic cross-clamp time, reflecting expected clinical correlation among operative-time variables. Therefore, the extended clinical model was interpreted as a supplementary adjustment model rather than the primary comparator. Detailed VIF results are presented in the Supplementary Appendix (Table S6).

### Component-specific and sensitivity analyses

Because the primary endpoint was clinically heterogeneous, we performed component-specific analyses in the temporal validation cohort. dIPC-RFI showed acceptable discrimination for major components with sufficient validation events, including CRRT, prolonged mechanical ventilation, neurological complications, and MODS component, with AUCs of 0.765, 0.790, 0.756, and 0.878, respectively. The combined model showed AUCs of 0.765, 0.831, 0.781, and 0.869 for these components, respectively. For components with fewer than five validation events, including death, IABP, ECMO, and tracheostomy, the analyses were considered exploratory and descriptive. Component-specific and key sensitivity analyses are summarized in Table 4.

**Table 4.** Component-specific and key sensitivity analyses in the temporal validation cohort.

Endpoint or sensitivity analysis	Total events	Validation N	Validation events	dIPC-RFI AUC	Combined AUC	Note
CRRT	46	99	10	0.765	0.765	Component-specific AUC in temporal validation cohort
Mechanical ventilation > =72 h	90	99	32	0.790	0.831	Component-specific AUC in temporal validation cohort
Neurological complications	32	99	10	0.756	0.781	Component-specific AUC in temporal validation cohort
MODS component	46	99	9	0.878	0.869	Component-specific AUC in temporal validation cohort
dIPC-RFI, excluding CRRT		89	34	0.879		Sensitivity analysis excluding CRRT patients
Combined, excluding CRRT		89	34		0.905	Sensitivity analysis excluding CRRT patients
Death or MODS component		99	10	0.875	0.873	Key severe endpoint sensitivity analysis

*Note:* Rare components with fewer than five validation events, including death, IABP, ECMO, and tracheostomy, are interpreted as exploratory/descriptive and are provided in the Supplementary Appendix.

The eight available component endpoints completely covered the primary composite outcome: 134 patients had at least one severe organ dysfunction component, and the union of component endpoints also identified 134 patients, with no discrepancy between the composite endpoint and the available component endpoints. After excluding patients who received CRRT, model performance remained robust. In the validation cohort excluding CRRT patients, dIPC-RFI achieved an AUC of 0.879 and a Brier score of 0.1428, whereas the combined model achieved an AUC of 0.905 and a Brier score of 0.1210. In an additional sensitivity analysis using death or MODS component as a key severe endpoint, dIPC-RFI achieved an AUC of 0.875 and the combined model achieved an AUC of 0.873. Component timing confirming that all events occurred after the 24-hour landmark is provided in the Supplementary Appendix (Table S7). Additional sensitivity analyses excluding CRRT or using death/MODS as the endpoint are presented in the Supplementary Appendix (Tables S8–S9).

### Decision curve analysis and clinical utility

**Figure 2.**
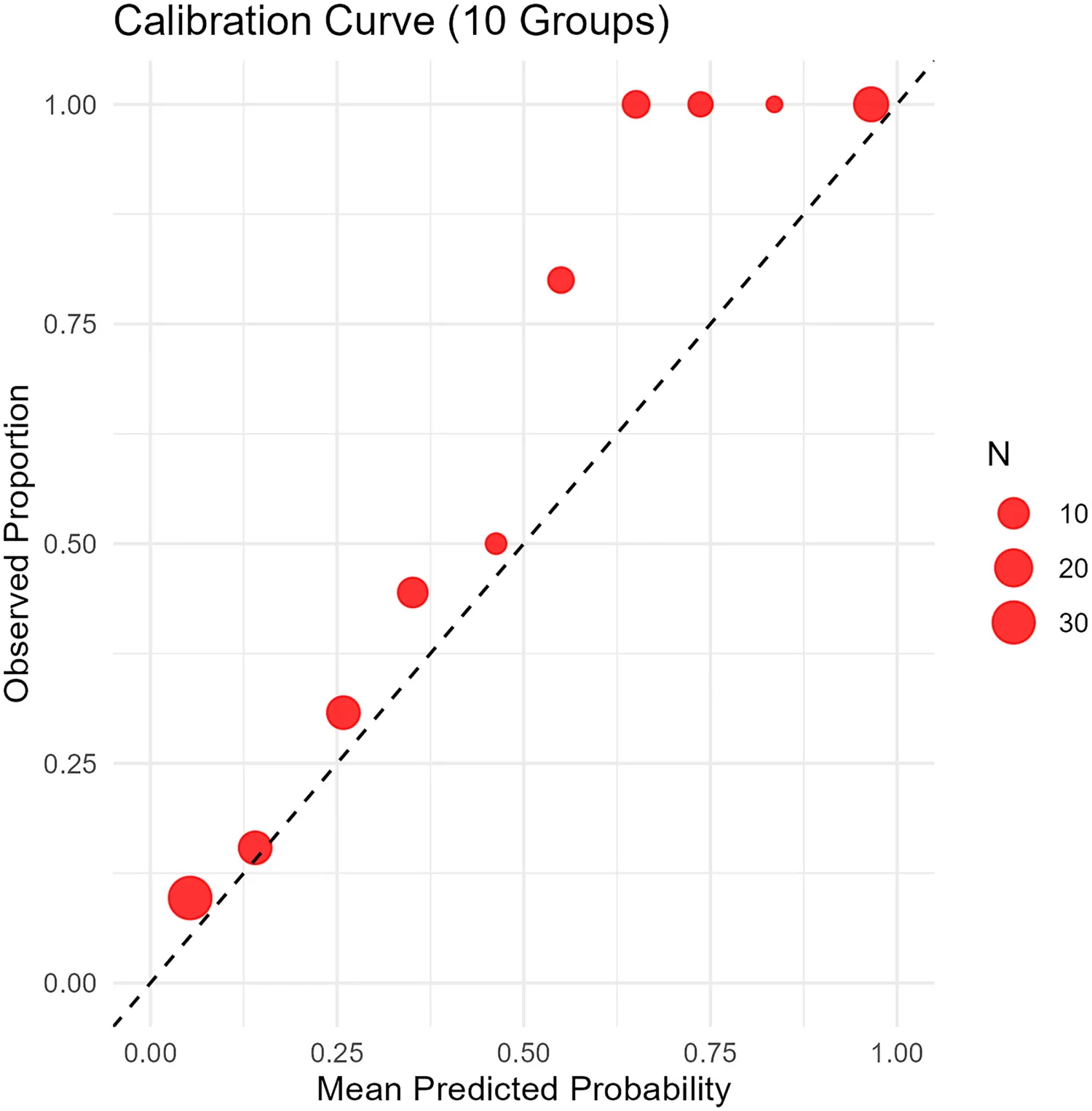
Calibration curve. The x-axis indicates mean predicted probability and the y-axis indicates observed event rate. The dashed line indicates perfect calibration, and bubble size indicates group sample size. The model showed good discrimination and acceptable calibration in the low-to-intermediate risk range, but may underestimate actual event risk in the high-risk range; external validation and recalibration may be necessary.

**Figure 3.**
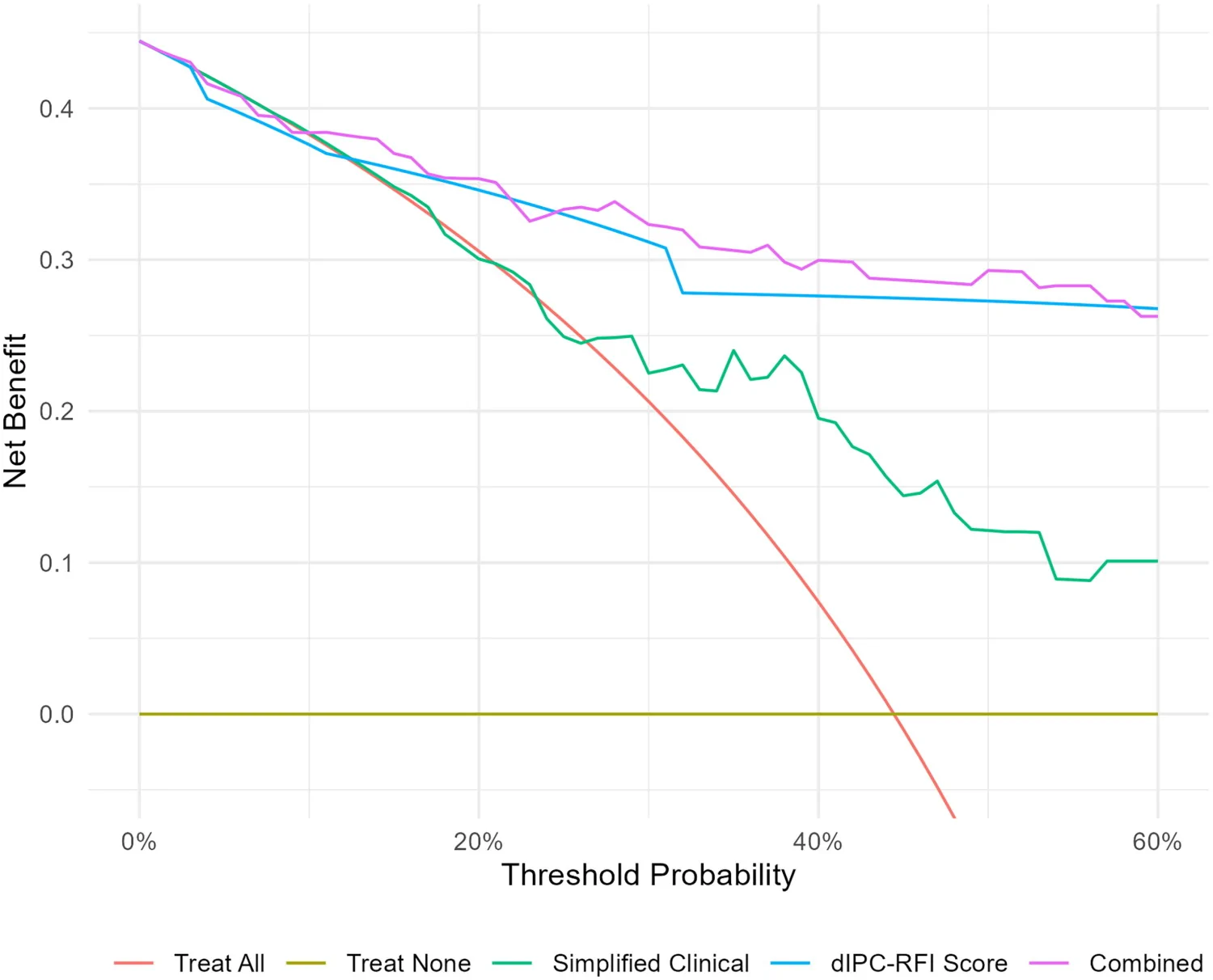
Decision curve analysis. The x-axis indicates threshold probability and the y-axis indicates net benefit. Treat all indicates intervention for all patients, and treat none indicates no intervention. dIPC-RFI and the combined model were superior to treat-all, treat-none, and the simplified clinical model over most thresholds, and the combined model showed greater net benefit in the clinically relevant threshold range of 0.10–0.40.

Decision curve analysis showed that the combined model generally provided greater net benefit than the simplified clinical model and dIPC-RFI alone across clinically relevant threshold probabilities, supporting its potential use for postoperative 24-hour risk restratification. The decision curve analysis for clinical net benefit across risk thresholds is displayed in Figure 3. The calibration curve is shown in Figure 2. These findings should be interpreted as evidence of risk-stratification value rather than proof that model-guided intervention improves outcomes.

Detailed results of missing data summary, risk strata, recovery-failure phenotypes, VIF assessments, component timing, and additional sensitivity analyses are presented in the Supplementary Appendix (Tables S1–S9).

## Discussion

### Main findings and clinical meaning

This single-center temporal validation study developed and evaluated dIPC-RFI as a postoperative 24-hour risk-restratification tool for patients undergoing surgery for ATAAD. The main finding is that early recovery failure across inflammation, perfusion, and coagulation systems identifies patients at increased risk of severe organ dysfunction occurring beyond 24 h after surgery. Unlike purely preoperative operative-risk scores, dIPC-RFI captures postoperative physiological response after surgical repair, cardiopulmonary bypass, resuscitation, transfusion, and early organ recovery. Therefore, its intended role is not preoperative surgical decision-making but structured ICU reassessment at a fixed postoperative 24-hour window.

The model retained clinically meaningful performance in the temporal validation cohort. dIPC-RFI outperformed the simplified clinical model, while the combined model further improved discrimination and reduced overall prediction error. The comparable performance of the continuous-variable model suggests that the point-based score did not sacrifice substantial predictive information. This is important for bedside use, because a simple score is easier to calculate, communicate, and integrate into postoperative ICU workflow than a multivariable continuous prediction equation.

### Relationship with SOFA and GERAADA

The relationship between dIPC-RFI and established scores deserves clarification. SOFA remains a widely used framework for describing established organ dysfunction in ICU patients. However, SOFA mainly quantifies the severity of organ dysfunction across multiple systems, whereas dIPC-RFI was designed to capture early postoperative recovery failure trajectories across inflammation, perfusion, and coagulation at a fixed 24-hour time point after ATAAD surgery. Therefore, dIPC-RFI should be viewed as complementary to SOFA rather than as a replacement.

In the present retrospective dataset, complete variables required to reliably reconstruct SOFA at exactly postoperative 24 h were not consistently available, particularly standardized neurological assessment and detailed vasoactive-drug information. We therefore did not perform a post hoc partial SOFA comparison, because incomplete reconstruction could introduce misclassification and generate a misleading comparison. Similarly, GERAADA is primarily intended for preoperative or perioperative mortality risk estimation in ATAAD, whereas dIPC-RFI is intended for postoperative risk restratification among patients who are alive and assessable at 24 h after surgery. Future prospective multicenter studies should directly compare dIPC-RFI with SOFA, GERAADA, and other established ICU or ATAAD risk scores to determine its incremental value and external generalizability.

### Composite endpoint heterogeneity

The primary outcome in this study was intentionally defined as a composite of clinically important severe organ dysfunction events beyond postoperative 24 h. This design reflects real-world postoperative ICU management after ATAAD surgery, where renal, respiratory, neurological, circulatory, and global organ failure events often overlap and may share upstream pathophysiological mechanisms, including systemic inflammation, hypoperfusion, ischemia-reperfusion injury, and coagulation disturbance. Nevertheless, the components differ in mechanism and severity. To address this limitation, we performed component-specific and sensitivity analyses. dIPC-RFI retained acceptable discrimination for major components with sufficient validation events, including CRRT, prolonged mechanical ventilation, neurological complications, and MODS component. The no-CRRT and death-or-MODS sensitivity analyses further suggested that the observed performance was not driven solely by renal replacement therapy or by a single component of the composite endpoint.

### Multicollinearity and model interpretation

The VIF analysis supports the stability of the continuous dIPC-RFI component model, because all VIF values were low and the maximum VIF was 1.574. In contrast, the extended clinical model showed expected multicollinearity involving operative-time variables, particularly cardiopulmonary bypass time and aortic cross-clamp time. These variables are clinically and mathematically related in ATAAD surgery and should not be interpreted as independent causal effects within the same model. For this reason, the extended clinical model was used as a supplementary adjustment analysis to test whether dIPC-RFI retained incremental risk-stratification value after accounting for perioperative bleeding, transfusion, and operative burden.

### Clinical application

Clinically, dIPC-RFI may help ICU teams identify patients who require different levels of postoperative attention at 24 h. Patients with low scores may continue standard ICU monitoring. Patients with intermediate scores may benefit from intensified reassessment of lactate, coagulation status, platelet trends, urine output, respiratory function, hemodynamics, and neurological status. Patients with high scores may require early multidisciplinary review, investigation for persistent hypoperfusion or bleeding/coagulation disturbance, and preparation for renal, respiratory, circulatory, or neurological support. Importantly, dIPC-RFI should be used as a risk-restratification aid, not as a stand-alone trigger for invasive organ support.

### Limitations

This study has several limitations. First, this was a single-centre retrospective study. Although temporal validation was performed by chronological split and postoperative 24 h was used as a fixed landmark to reduce temporal ambiguity, external validation in independent multicenter cohorts remains necessary. Second, the primary outcome was a heterogeneous composite endpoint. Although all component events occurred after the 24-hour dIPC-RFI assessment and component-specific analyses were performed, some components had few events in the temporal validation cohort. Therefore, analyses for death, IABP, ECMO, and tracheostomy should be interpreted as exploratory and descriptive rather than confirmatory. Third, the cutoffs used in dIPC-RFI were derived using the Youden index in the derivation cohort. Although these cutoffs were fixed before temporal validation, they remain data-derived and should be considered provisional until independently validated in external cohorts.

Fourth, the primary analysis was based on patients with complete variables required for dIPC-RFI assessment at postoperative 24 h. This complete-case approach was necessary for score construction but may introduce selection bias if patients with missing required serial variables differed systematically from those included in the analysis. Future prospective studies should use predefined data collection to minimize missingness and evaluate the impact of missing data. Fifth, complete variables required to reliably reconstruct SOFA at exactly postoperative 24 h were not consistently available, and complete GERAADA variables were also unavailable. We therefore did not perform incomplete post hoc score reconstruction. Future studies should include standardized SOFA, GERAADA, and dIPC-RFI assessment to permit direct head-to-head comparison. Sixth, the extended clinical model showed expected multicollinearity among operative-time variables; therefore, it should be interpreted as a supplementary adjustment model rather than as a definitive causal model. Finally, this study evaluated risk stratification but did not test whether dIPC-RFI-guided interventions improve outcomes. The score should not replace clinical judgment or serve as a stand-alone indication for invasive organ support.

## Conclusions

dIPC-RFI provides a simple 24-hour postoperative framework for identifying incomplete recovery across inflammation, perfusion, and coagulation after surgery for acute type A aortic dissection. In the temporal validation cohort, higher dIPC-RFI scores identified patients at substantially increased risk of severe organ dysfunction beyond 24 h. Clinically, the score may help ICU teams select patients who require intensified monitoring, repeated lactate and coagulation assessment, early multidisciplinary review, and preparation for renal, respiratory, circulatory, or neurological support. dIPC-RFI should be used as a risk-restratification aid rather than a stand-alone treatment trigger, and prospective multicentre validation is required before routine implementation.

### dIPC-RFI calculator

A simple dIPC-RFI calculator was developed to allow entry of postoperative 24-hour NLR, preoperative NLR, postoperative 24-hour lactate, immediate postoperative lactate, postoperative 24-hour INR, and preoperative and postoperative 24-hour platelet counts. The calculator automatically generates the dIPC-RFI total score and risk stratum. Link: https://gorgeous-cobbler-5d3ca7.netlify.app/Calculator QR code
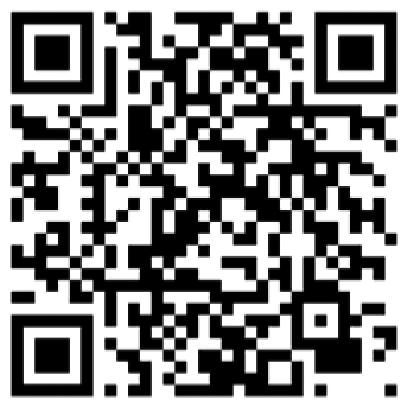


The calculator does not store patient data and is intended only for research demonstration and bedside reference. It cannot replace clinical judgment. If the calculator or QR code cannot be obtained, you can contact the corresponding author.

## Supplementary Material

Supplemental Material

## Data Availability

The data that support the findings of this study are available from the corresponding author upon reasonable request. The data are not publicly available because they contain information that could compromise the privacy of research participants.
