# Supplementary material for: A dynamic recovery failure index after acute type A aortic dissection surgery
Source: Ann Med. 2026 Sep 4;58(1):2724602. doi: 10.1080/07853890.2026.2724602 (PMC13552331; doi:10.1080/07853890.2026.2724602)
Supplement: Supplementary file 1 [file IANN_A_2724602_SM7062.docx]

### Table S1. Missing data summary of key variables included in the study

| Variable | N_total | N_missing | Missing_percent | N_complete |
| --- | --- | --- | --- | --- |
| Sex | 330 | 0 | 0% | 330 |
| Age | 330 | 0 | 0% | 330 |
| Body mass index (BMI) | 330 | 0 | 0% | 330 |
| Postoperative continuous renal replacement therapy (CRRT) | 330 | 0 | 0% | 330 |
| Cardiopulmonary bypass time | 330 | 0 | 0% | 330 |
| Aortic cross-clamp time | 330 | 0 | 0% | 330 |
| Operative duration | 330 | 0 | 0% | 330 |
| Primary outcome | 330 | 0 | 0% | 330 |
| Neutrophil-to-lymphocyte ratio (NLR) at preoperative assessment | 330 | 0 | 0% | 330 |
| NLR at postoperative 24 hours | 330 | 0 | 0% | 330 |
| Lactate at preoperative assessment | 330 | 0 | 0% | 330 |
| Lactate immediately postoperatively | 330 | 0 | 0% | 330 |
| Lactate at postoperative 24 hours | 330 | 0 | 0% | 330 |
| International normalized ratio (INR) at postoperative 24 hours | 330 | 0 | 0% | 330 |
| Mechanical ventilation ≥72 h | 330 | 0 | 0% | 330 |
| Neurological complications | 330 | 0 | 0% | 330 |
| Multiple organ dysfunction syndrome (MODS) component | 330 | 0 | 0% | 330 |
| Death after 24 h / 30-day death | 330 | 0 | 0% | 330 |
| Intra-aortic balloon pump (IABP) | 330 | 0 | 0% | 330 |
| Extracorporeal membrane oxygenation (ECMO) | 330 | 0 | 0% | 330 |
| Tracheostomy | 330 | 0 | 0% | 330 |

**Note:** All variables required for dIPC-RFI scoring and primary outcome definition had 0% missingness due to the complete-case inclusion criteria of the study.

### Table S2. Risk strata distribution in derivation and temporal validation cohorts

| Cohort | Risk group | N | Events | Event rate (%) |
| --- | --- | --- | --- | --- |
| Derivation | Low (score 0–1) | 104 | 7 | 6.7% |
| Derivation | Intermediate (score 2–3) | 70 | 32 | 45.7% |
| Derivation | High (score 4–6) | 57 | 51 | 89.5% |
| Temporal validation | Low (score 0–1) | 41 | 5 | 12.2% |
| Temporal validation | Intermediate (score 2–3) | 43 | 24 | 55.8% |
| Temporal validation | High (score 4–6) | 15 | 15 | 100.0% |

**Note:** Risk groups were defined as low (0–1 point), intermediate (2–3 points), and high (4–6 points) based on the dIPC-RFI score.

### Table S3. Recovery-failure phenotypes by number of affected systems

| Cohort | Phenotype | N | Events | Event rate (%) |
| --- | --- | --- | --- | --- |
| Derivation | Recovery-preserved phenotype | 55 | 1 | 1.8% |
| Derivation | Single-system recovery failure | 66 | 12 | 18.2% |
| Derivation | Dual-system recovery failure | 63 | 36 | 57.1% |
| Derivation | Three-system recovery failure | 47 | 41 | 87.2% |
| Temporal validation | Recovery-preserved phenotype | 14 | 2 | 14.3% |
| Temporal validation | Single-system recovery failure | 46 | 11 | 23.9% |
| Temporal validation | Dual-system recovery failure | 25 | 17 | 68.0% |
| Temporal validation | Three-system recovery failure | 14 | 14 | 100.0% |

**Note:** Phenotypes were defined based on the number of abnormal domains among inflammation (NLR at 24 h or delta NLR), perfusion (lactate at 24 h or lactate clearance), and coagulation (INR at 24 h or platelet decline rate).

### Table S4. Cutoffs for the unified rate-of-change version (dIPC-RFI-rate) derived in the derivation cohort

**Table S4. Cutoffs for the unified rate-of-change version (dIPC-RFI-rate) derived in the derivation cohort**

| Variable | Cutoff |
| --- | --- |
| NLR at postoperative 24 hours | 11.731 |
| NLR change rate from preoperative to postoperative 24 hours (%) | 0.135 |
| Lactate at postoperative 24 hours (mmol/L) | 2.350 |
| Lactate change rate from preoperative to postoperative 24 hours (%) | 2.000 |
| INR at postoperative 24 hours | 1.560 |
| Platelet decline rate from preoperative to postoperative 24 hours (%) | 46.174% |

**Note:** The unified change rate was defined as (value at postoperative 24 hours − value at preoperative assessment) / value at preoperative assessment × 100% for variables where higher values indicated higher risk. For platelet decline, the rate was defined as (platelet count at preoperative assessment − platelet count at postoperative 24 hours) / platelet count at preoperative assessment × 100% to reflect the direction of increased risk. dIPC-RFI-rate was constructed as a sensitivity analysis to evaluate the influence of dynamic-variable definitions on model performance.

### Table S5. DeLong test comparison between dIPC-RFI and dIPC-RFI-rate in the temporal validation cohort

| Comparison | AUC (dIPC-RFI) | AUC (rate) | Difference (rate − original) | P value | 95% CI (lower) | 95% CI (upper) |
| --- | --- | --- | --- | --- | --- | --- |
| dIPC-RFI vs dIPC-RFI-rate | 0.873 | 0.885 | 0.012 | 0.5041 | −0.0471 | 0.0232 |

**Note:** The DeLong test showed no statistically significant difference between the original dIPC-RFI and the unified rate-of-change version, suggesting that the model performance was robust to the definition of dynamic change variables.

### Table S6. Variance inflation factor (VIF) details for multicollinearity assessment

**Table S6a. VIF for the continuous-variable model**

| Model | Variable | VIF | N_complete |
| --- | --- | --- | --- |
| Continuous-variable model | NLR at postoperative 24 hours | 1.347 | 231 |
| Continuous-variable model | Delta NLR (postoperative 24 h − preoperative) | 1.450 | 231 |
| Continuous-variable model | Lactate at postoperative 24 hours (mmol/L) | 1.574 | 231 |
| Continuous-variable model | Lactate clearance (immediate postoperative → 24 h, %) | 1.269 | 231 |
| Continuous-variable model | INR at postoperative 24 hours | 1.207 | 231 |
| Continuous-variable model | Platelet decline rate (preoperative → 24 h, %) | 1.125 | 231 |

**Note:** All VIF values were below the conventional threshold of 5, indicating no substantial multicollinearity among the six continuous dIPC-RFI components.

**Table S6b. VIF for the extended clinical model (before and after addition of dIPC-RFI)**

| Model | Variable | VIF | N_complete |
| --- | --- | --- | --- |
| Extended clinical model | Age | 1.145 | 231 |
| Extended clinical model | Sex | 1.140 | 231 |
| Extended clinical model | Body mass index (BMI) | 1.083 | 231 |
| Extended clinical model | Cardiopulmonary bypass time | 8.173 | 231 |
| Extended clinical model | Aortic cross-clamp time | 5.126 | 231 |
| Extended clinical model | Operative duration | 3.831 | 231 |
| Extended clinical + dIPC-RFI model | Age | 1.157 | 231 |
| Extended clinical + dIPC-RFI model | Sex | 1.140 | 231 |
| Extended clinical + dIPC-RFI model | BMI | 1.084 | 231 |
| Extended clinical + dIPC-RFI model | Cardiopulmonary bypass time | 8.501 | 231 |
| Extended clinical + dIPC-RFI model | Aortic cross-clamp time | 5.205 | 231 |
| Extended clinical + dIPC-RFI model | Operative duration | 3.832 | 231 |
| Extended clinical + dIPC-RFI model | dIPC-RFI score | 1.112 | 231 |

**Table S6c. VIF summary interpretation**

| Model | Max VIF | Variables with VIF > 5 | Interpretation |
| --- | --- | --- | --- |
| Continuous-variable model | 1.574 | None | No substantial multicollinearity (all VIF < 5) |
| Extended clinical model | 8.173 | Cardiopulmonary bypass time; Aortic cross-clamp time | Expected multicollinearity among operative-time variables |
| Extended clinical + dIPC-RFI model | 8.501 | Cardiopulmonary bypass time; Aortic cross-clamp time | Expected multicollinearity; interpret supplementary adjustment model cautiously |

### Table S7. Timing of component endpoints relative to postoperative 24-hour landmark

**Table S7a. All component events occurred after postoperative 24 hours**

| Outcome component | Total events | Events before postoperative 24 h | Events after postoperative 24 h |
| --- | --- | --- | --- |
| CRRT | 46 | 0 | 46 |
| Mechanical ventilation ≥72 h | 90 | 0 | 90 |
| Neurological complications | 32 | 0 | 32 |
| MODS component | 46 | 0 | 46 |
| Death after 24 h / 30-day death | 17 | 0 | 17 |
| IABP | 3 | 0 | 3 |
| ECMO | 9 | 0 | 9 |
| Tracheostomy | 10 | 0 | 10 |
| Patients with any severe organ dysfunction | 134 | 0 | 134 |

**Table S7b. Union of component endpoints completely covered the primary composite outcome**

| Item | Count |
| --- | --- |
| Primary composite outcome events | 134 |
| Union of available component endpoints | 134 |
| Difference | 0 |

**Note:** The union of the eight component endpoints completely covered the primary composite outcome, confirming that no primary outcome event was missing from the component-specific analyses.

### Table S8. Sensitivity analysis excluding patients who received CRRT

| Model | Validation N | Events | AUC (95% CI) | Brier score (95% CI) | Calibration intercept (95% CI) | Calibration slope (95% CI) |
| --- | --- | --- | --- | --- | --- | --- |
| Simplified clinical, excluding CRRT | 89 | 34 | 0.777 (0.659–0.875) | 0.1953 (0.1537–0.2407) | 1.349 (0.500–2.789) | 1.940 (1.120–3.547) |
| dIPC-RFI, excluding CRRT | 89 | 34 | 0.879 (0.801–0.945) | 0.1428 (0.0992–0.1908) | 1.392 (0.651–3.234) | 1.600 (1.000–3.114) |
| Combined, excluding CRRT | 89 | 34 | 0.905 (0.828–0.968) | 0.1210 (0.0771–0.1692) | 1.153 (0.459–2.356) | 1.315 (0.908–2.449) |

**Note:** In this sensitivity analysis, patients who received CRRT were excluded to assess whether the model performance was driven solely by renal replacement therapy. dIPC-RFI retained robust discrimination (AUC = 0.879) and the combined model further improved performance (AUC = 0.905).

### Table S9. Sensitivity analysis using death or MODS component as a key severe endpoint

| Endpoint | Validation N | Validation events | dIPC-RFI AUC | Combined AUC |
| --- | --- | --- | --- | --- |
| Death or MODS component | 99 | 10 | 0.875 | 0.873 |

**Note:** In this sensitivity analysis, the endpoint was defined as death after postoperative 24 hours or MODS component, representing the most clinically severe outcomes. Both dIPC-RFI and the combined model maintained good discrimination (AUC > 0.85).
